# Galectin-3: The Impact on the Clinical Management of Patients with Thyroid Nodules and Future Perspectives

**DOI:** 10.3390/ijms19020445

**Published:** 2018-02-02

**Authors:** Armando Bartolazzi, Salvatore Sciacchitano, Calogero D’Alessandria

**Affiliations:** 1 Pathology Research Laboratory, Saint Andrea University Hospital, via di Grottarossa 1035, 00189 Rome, Italy; 2 Department of Clinical and Molecular Medicine, Sapienza University, Policlinico Umberto I viale Regina Elena 324, 00161 Rome, Italy; Salvatore.Sciacchitano@uniroma1.it; 3 Laboratory of Biomedical Research, Niccolò Cusano University Foundation, via Don Carlo Gnocchi 3, 00166 Rome, Italy; 4 Nuklearmedizinische Klinik und Poliklinik, Klinikum rechts der Isar, Technische Universität München, Ismaninger Strasse 22, 81675 München, Germany; Calogero.dalessandria@tum.de

**Keywords:** galectin-3, thyroid cancer, thyroid FNA-cytology, tumor imaging in vivo, immuno-PET

## Abstract

Galectins (S-type lectins) are an evolutionarily-conserved family of lectin molecules, which can be expressed intracellularly and in the extracellular matrix, as well. Galectins bind β-galactose-containing glycoconjugates and are functionally active in converting glycan-related information into cell biological programs. Altered glycosylation notably occurring in cancer cells and expression of specific galectins provide, indeed, a fashionable mechanism of molecular interactions able to regulate several tumor relevant functions, among which are cell adhesion and migration, cell differentiation, gene transcription and RNA splicing, cell cycle and apoptosis. Furthermore, several galectin molecules also play a role in regulating the immune response. These functions are strongly dependent on the cell context, in which specific galectins and related glyco-ligands are expressed. Thyroid cancer likely represents the paradigmatic tumor model in which experimental studies on galectins’ glycobiology, in particular on galectin-3 expression and function, contributed greatly to the improvement of cancer diagnosis. The discovery of a restricted expression of galectin-3 in well-differentiated thyroid carcinomas (WDTC), compared to normal and benign thyroid conditions, contributed also to promoting preclinical studies aimed at exploring new strategies for imaging thyroid cancer in vivo based on galectin-3 immuno-targeting. Results derived from these recent experimental studies promise a further improvement of both thyroid cancer diagnosis and therapy in the near future. In this review, the biological role of galectin-3 expression in thyroid cancer, the validation and translation to a clinical setting of a galectin-3 test method for the preoperative characterization of thyroid nodules and a galectin-3-based immuno-positron emission tomography (immuno-PET) imaging of thyroid cancer in vivo are presented and discussed.

## 1. The Clinical Problem of the Preoperative Characterization of Follicular Thyroid Nodules

It has been estimated that as many as 5–6% of the adult population in the USA (about 15 million people) have clinically-evident thyroid nodules, but this number is much higher (up to 65%) if sub-clinical nodules incidentally discovered during a thyroid echo-scan are also counted [1,2]. Distinguishing among benign and malignant thyroid nodules is a challenge. In fact, it is very difficult to detect preoperatively a relatively rare thyroid cancer (about 12–15% of total thyroid nodules) among a multitude of benign thyroid nodules [3,4,5,6,7,8,9,10,11,12]. The wide use of thyroid fine-needle-aspiration (FNA) cytology contributed to improving the preoperative characterization of thyroid nodules, allowing a better surgical selection of these lesions and increasing, at the same time, the number of thyroid cancers diagnosed at histology.

**Figure 1:**
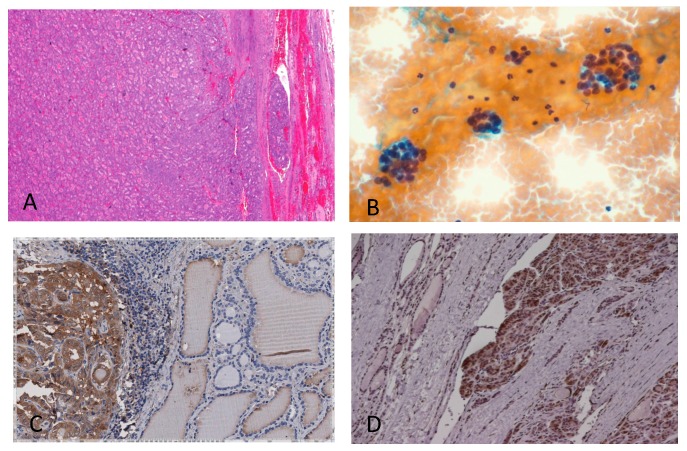
Follicular thyroid lesions and galectin-3 expression on histological samples. (**A**) Histological features of follicular carcinoma showing capsular and vascular infiltration. (**B**) This features cannot be detected on cytological bases. Fine-needle-aspiration-derived thyrocytes arranged in follicular structures remain indeterminate (defined as category Thy-3f according to the British Thyroid Association). (**C**) Galectin-3 expression on a histological sample of the follicular variant of papillary thyroid carcinoma. (**D**) Galectin-3 expression on follicular thyroid carcinoma with capsular infiltration. Magnification: (**A**) ×200; (**B**–**D**) ×250. (**A**,**B**) Conventional haematoxylin/eosin staining; (**C**,**D**) direct immunoperoxidase staining by using a horseradish-peroxidase-conjugated (HRP) monoclonal antibody to galectin-3.

Thyroid FNA cytology was first proposed at Radiumhemmet, Karolinska Hospital, Solna (Sweden), during the year 1950 with the main purpose of distinguishing preoperatively among benign and malignant thyroid proliferations. The first report was published in Acta Medica Scandinava in 1952 [13]. Actually, thyroid FNA cytology is widely accepted as the most effective procedure for the preoperative diagnosis of thyroid cancer. However, since the first experience with the method, two major limitations were observed: the first is linked to the adequacy of the sampling, although it can be presently improved by performing FNA with an ultrasonographic guidance; the second limitation, which occurs in 15–30% of the cases (depending on the diagnostic thyroid center considered), represents, instead, an intrinsic limit of thyroid-cytology in providing a correct morphological diagnosis of benign (hyperplasia and adenoma) and malignant follicular thyroid lesions (follicular carcinoma, oncocytic follicular carcinoma and follicular variant of papillary carcinoma). This task is practically impossible on cytological bases alone due to the overlapping cyto-morphological features in benign and malignant follicular thyroid lesions [14,15,16,17,18,19,20,21,22]. In particular, the diagnosis of follicular carcinoma requires unequivocal demonstration of capsular and/or vascular invasion. These specific morphological hallmarks cannot be observed on cytological bases (Figure 1).

Follicular thyroid nodules at conventional FNA cytology remain, indeed, indeterminate and are classified as Thy-3 according to the British Thyroid Association, or Diagnostic Category III: AUS/FLUS (atypia of undetermined significance/follicular lesion of undetermined significance) according to the Bethesda System for thyroid cytology classification [23,24]. As a consequence, patients bearing cytologically-indeterminate follicular nodules are frequently referred to surgery for a partial or complete thyroidectomy, more for diagnosis rather than for a real therapeutic necessity. As expected, 85–90% of these lesions will be classified as benign at the final histology, and a large proportion of patients will be over-treated [3,15,16,17,25,26,27,28].

Although the current clinical management of patients with thyroid nodules seems to be “methodologically efficient”, considering the relatively low incidence of thyroid cancer and its low mortality rate, the surgical overtreatment of such a large number of patients with benign nodules represents a social problem. In Germany, for example, a country with a high prevalence of follicular thyroid nodules, around 100,000 thyroid surgical procedures for benign thyroid lesions are registered each year [29]. The large majority of these lesions are likely benign follicular nodules that were not fully-characterized preoperatively.

In addition to the relevant costs for the public health system and patients’ distress, people undergoing surgery for thyroid nodules have a low, but significant risk of permanent injury of the parathyroid glands and recurrent nerves. Life-long substitutive thyroid hormone therapy is always necessary, and calcium supplementation may be also required for patients with impaired (iatrogenic) parathyroid function.

In the last two decades, many efforts have been directed toward developing new potential diagnostic tools for improving thyroid cancer diagnosis preoperatively, and galectin-3 is probably one of the most extensively-studied marker so far. The expression analysis of galectin-3 in thyroid lesions, applied preoperatively for improving the diagnostic performance of conventional FNA cytology, has been finally validated for translation to the clinical setting. Two large multi-institutional studies performed at the international level and involving university hospitals and specialized thyroid centers contributed to this achievement [30,31]. Since the year 1995 when a preliminary report on galectin-3 and thyroid cancer was published [32], despite a great variance in the methodology used, more than 300 papers published in the English literature confirmed the restricted expression of galectin-3 in thyroid cancer, compared to normal and benign thyroid conditions [33,34,35,36,37,38,39,40,41]. Moreover, an independent study of the largest thyroid cancer diagnostic marker panel reported to date showed that galectin-3 was the most accurate stand-alone marker for well-differentiated thyroid cancer, compared to 56 different candidate molecules [42,43]. Altogether, these data strongly support the potential role of galectin-3 as a reliable diagnostic marker for thyroid cancer diagnosis.

## 2. The Biological Rationale of Galectin-3 Expression in Transformed Thyroid Cells

Galectin-3 is almost invariably expressed in well-differentiated thyroid carcinomas and can be promptly detected in the cytoplasm of malignant thyroid cells by using immunohistochemical procedures. On the other side, galectin-3 is undetectable in the cytoplasm of normal thyroid follicular cells. This diagnostically-relevant finding has been extensively confirmed in the literature in experimental tumor models in vitro, as well as on cyto-histological substrates ex vivo [30,31,32,33,34,35,36,37,38,39,40,41,42,43].

By using an in vitro model of normal thyroid cells named TAD-2, Takenaka et al. demonstrated that a forced expression of galectin-3 via cDNA transfection induced a transformed phenotype [44]. Stable galectin-3-positive TAD-2 transfectants, in fact, acquired the phenotype of serum-independent growth, clonogenicity in soft agar and loss of contact inhibition. Moreover, a gene expression profile performed on galectin-3 transfectants revealed activation of genes involved in tumor growth and progression, among which were PCNA (proliferating cell nuclear antigen), replication factor C and *Rb* retinoblastoma gene. The latter’s protein product plays a significant role in G1–S transition. Conversely, in a different set of experiments, which used a thyroid cancer and a breast carcinoma cell line, inhibition of galectin-3 expression by using mRNA interference reverted the transformed phenotype [45,46].

These experimental findings clearly demonstrate that galectin-3 likely plays a relevant biological role in thyroid cancer. The aberrant expression of galectin-3 in normal thyroid cells, in fact, blocks the apoptotic program, allowing accumulation of DNA mutations and molecular alterations, which in turn promote the development of cancer.

The galectin-3 COOH-terminal domain contains an NWGR amino acid motif highly conserved in the BH1 domain of the Bcl-2 family of anti-apoptotic molecules. The NWGR amino acid sequence is critical for regulating apoptosis as demonstrated by experimental studies in vitro, which used cell transfectants carrying glycine to alanine substitution in the NWGR motif, exposed to *cis*-platinum (CDDP), a potent anticancer compound that produces an interstrand DNA cross-link and induces apoptosis. Galectin-3 mutant transfectants in the NWGR motif showed high sensitivity to CDDP exposure in vitro compared to the control cell lines expressing wild-type galectin-3 that remain largely viable [47]. More recently, it has been reported that galectin-3 is a physiological target of p53 transcriptional activity. A p53-dependent down-regulation of galectin-3 expression, occurring at transcriptional level, is required for triggering the p53-mediated apoptotic program in different cell systems [48]. This means that following DNA damage, wild-type p53 does not work properly in activating the apoptotic program in a cell context in which galectin-3 remains upregulated. Indeed, in well-differentiated thyroid carcinoma (WDTC) that notably express wt-p53, an unexplained paradoxical concomitant expression of galectin-3 seems to occur. Interestingly, a loss of p53 activator HIPK2 (homeodomain interacting protein kinase-2), a critical molecule that is necessary for p53 phosphorylation on serine 46, has been finally demonstrated in WDTC and was found responsible for p53 loss of function, galectin-3 overexpression and block of apoptosis [49]. In line with these findings, genetic studies also show that a hypomethylation state of 5 CpG sites in the galectin-3 gene correlated with thyroid malignancies [50].

All together, these findings provide a strong biological rationale for the restricted expression of galectin-3 in malignant thyroid cells compared to normal and benign thyroid conditions. Furthermore, a plethora of experimental data published in the literature definitively demonstrates that WDTC almost invariably expresses galectin-3, while normal thyroid tissue, follicular nodular hyperplasia (multinodular goiters) and the large majority of thyroid follicular adenomas do not [33,34,35,36,37,38,39,40,41,42,43,51].

## 3. Validation of a Galectin-3 Test Method for Clinical Use

With this biological background, the potential diagnostic value of galectin-3 expression analysis in distinguishing among benign and malignant thyroid nodules has been deeply investigated in a large retrospective international multicenter study, which included institutions from Italy, Sweden, the United States and Japan [30]. In this study, as many as 1006 retrospective and histologically well-characterized thyroid lesions were independently analyzed at the immunohistochemical level for galectin-3 expression. The analysis used a purified and well-characterized mAb to galectin-3. Sensitivity, specificity, positive predictive value and diagnostic accuracy of galectin-3 expression in distinguishing among benign and malignant thyroid lesions were 99%, 98%, 91% and 97%, respectively, demonstrating that galectin-3 expression analysis is a potent and reliable diagnostic tool for thyroid cancer detection ex vivo [30].

A galectin-3 test-method optimized for clinical use was applied, indeed, on cytological substrates in a prospective large multicenter study, which involved 11 thyroid institutions and cancer centers. In this study, carried out on 466 patients bearing Thy-3 follicular thyroid proliferations as candidates for surgery, galectin-3 expression analysis was applied preoperatively on FNA-derived cellblock preparations by using immunocyto-histochemistry [31].

The final centralized histological characterization of the resected follicular thyroid lesions performed in blind by two independent and eminent pathologists confirmed that galectin-3 expression analysis is an inexpensive and useful diagnostic tool, which can be easily used in clinical practice for improving the diagnostic performance of conventional thyroid FNA cytology. The sensitivity and specificity of the test-method applied preoperatively were 78% and 93%, respectively. Most importantly, 88% of the Thy-3 follicular thyroid proliferations referred for surgery were correctly classified preoperatively by using the galectin-3 test method alone [31].

Results of these studies are very exciting from the clinical point of view, considering the fact that the correct preoperative application of the test method allows one to avoid up to 71% of unnecessary thyroid surgical procedures, in a clinical scenario in which almost all of the follicular thyroid proliferations (with indeterminate cytology) are still referred for surgery. Moreover, when the galectin-3 test method is correctly used in a clinical-pathological multidisciplinary context its diagnostic performance is further improved [52].

**Figure 2:**
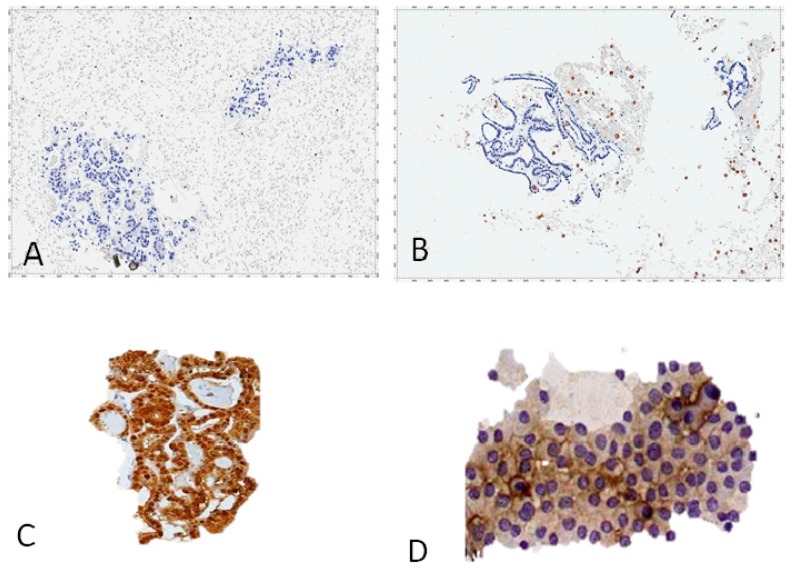
Cellblocks’ preparations from thyroid FNA cytology stained with an HRP-conjugated mAb to galectin-3. Benign follicular thyroid proliferations do not express galectin-3: (**A**) follicular adenoma; (**B**) nodular hyperplasia (gal-3-positive scattered foamy macrophages serve as the internal positive control). Thyroid malignancies expressing galectin-3: (**C**) papillary carcinoma follicular variant; (**D**) follicular carcinoma; (**A**–**D**) direct immunoperoxidase staining by using an HRP-conjugated mAb to gal-3. Magnification: ×250.

Presently, an optimized galectin-3 test method for cyto-histological use has been translated to the clinical setting and is routinely used in many thyroid institutions worldwide (Figure 2) [53].

Since the year 2003, galectin-3 expression analysis has been mentioned in the American Thyroid Association Guidelines for the clinical management of thyroid nodules [54] and more recently in the revised work [55]. As highlighted by Trimboli et al. in their contribution to this special issue [56], the galectin-3 test method performs better on histological substrates compared to FNA-derived cytological preparations. This is clearly expected if we consider the diagnostic and technical variability that typically affects cytology and immunocytochemistry. In our experience, there is still space for improving FNA-derived cytological preparations, immunocyto-histochemical staining and interpretation of galectin-3 results on FNA-derived thyroid cellblocks. Basic technical and operative guidelines for the optimal diagnostic performance of the galectin-3 test method have been published in order to avoid the occurrence of false negative and false positive results [54]. The most important technical requirements for a reliable galectin-3 test method are summarized in Table 1.

When galectin-3 expression analysis is used for diagnostic purposes, the specific demonstration of galectin-3 accumulation in the cytoplasm of transformed follicular thyroid cells is imperative, independently of the presence or not of positive nuclear staining. This finding is biologically relevant because it is the accumulation of galectin-3 in the cytoplasm that triggers the anti-apoptotic function. With this in mind, we can write with confidence that a correct and reliable biological characterization of a thyroid lesion always requires a combined morphological and immuno-phenotypical approach. For this reason, galectin-3 expression analysis does not replace the conventional thyroid FNA cytology, but integrates this method, with the final result of a consistent improvement of the diagnostic performance [31].

The clinical problem of the preoperative characterization of thyroid nodules is a challenge, and since the publication of the first multicenter study in which galectin-3 expression analysis was applied on thyroid histological samples [30], several molecular approaches including gene expression profile [57] and mutational analysis [58] were also proposed for resolving this important clinical problem. Recently, a comprehensive comparative study on the diagnostic performance, feasibility and cost effectiveness of several diagnostic test methods, including the molecular methods proposed for thyroid cancer diagnosis, has been published [59].

Surprisingly galectin-3 expression analysis applied at the immunocyto-histochemical level on paraffin-embedded cyto-histological substrates showed a better diagnostic performance compared to the GEC and Afirma molecular tests. Galectin-3 serves well both as an efficient rule-out and rule-in test method, with a good likelihood ratio and diagnostic accuracy. Furthermore, and very importantly, galectin-3 expression analysis does not require centralization in a single specialized laboratory, but can be easily performed in each conventional laboratory of histology [59].

At this point, objective considerations concerning the cost of these diagnostic test methods are also necessary. The cost of galectin-3 immunohistochemical analysis is about 113 US Dollars/test and indeed is very competitive if compared with the cost estimated for the molecular genetic tests (20-times more expensive). For this reason, the galectin-3 test-method has a potential screening role [58].

Considering the prevalence of indeterminate thyroid nodules at conventional cytology (about 15–30% of FNA specimens), the cost savings offered by a galectin-3-based immunocyto-histological assay would result in being significant for both low income countries and industrialized countries where specialized thyroid hospitals examine thousands of patients per year.

## 4. Thyroid Cancer Imaging In Vivo by Targeting Galectin-3

As previously mentioned, the high prevalence of benign thyroid nodules in the adult population and the relatively rare occurrence of thyroid cancer make the preoperative identification of malignant nodules very difficult. Several imaging approaches are currently used in clinical practice with the attempt to detect thyroid cancer, but unfortunately, these methods are not specific enough.

Thyroid scintigraphy with radioiodine, for example, is widely used preoperatively in patients with one or more thyroid nodules. The method provides functional information on iodine uptake (cold or hot nodules), but it fails to distinguish among benign and malignant thyroid nodules.

On the other side, positron emission tomography (PET) in combination with different PET tracers like [^18^F]-2-fluoro-2-deoxy-d-glucose (^18^F-FDG), ^18^F-DOPA and ^68^Ga-somatostatin analogues has been also used for the same purpose [60]. In particular ^18^F-FDG PET has been proposed as a preoperative diagnostic procedure for detecting thyroid cancer. Although a cancer diagnosis is generally ruled out in the presence of negative ^18^F-FDG PET, the sensitivity and specificity of the method are not optimal [61,62,63]. Considering the plethora of data derived by the extensive analysis of galectin-3 expression in normal, benign and malignant thyroid tissue and the aforementioned biological rationale of galectin-3 expression in transformed thyroid cells, the idea to image thyroid cancer in vivo by targeting galectin-3 seems to be promising, coherent and clinically relevant.

Our group first proposed a novel approach for imaging thyroid cancer in vivo based on galectin-3 immunotargeting [64]. A preliminary set of experiments was performed on human thyroid cancer cell lines xenografted in a murine experimental model. A galectin-3-based thyroid immunoscintigraphy, which used as tracer a ^99m^Tc-labeled mAb to galectin-3 and as detector a prototype of a mini-γ camera for small animal imaging, was used ad hoc for imaging thyroid cancer xenografts in vivo. Results from this preliminary study showed a good and reliable imaging of galectin-3-positive thyroid tumors between 6 and 9 h from injection of 100 µCi of radiotracer in the tail vein, opening a new avenue in thyroid cancer diagnosis [64]. These preliminary results clearly show the real possibility of detecting thyroid cancer in vivo by targeting galectin-3.

Recently, a galectin-3-based immuno-positron emission tomography (immuno-PET) for imaging thyroid cancer in vivo has been developed and used in preclinical experimental models of thyroid cancer xenografts. The method used a thyroid cancer-specific probe obtained by radiolabeling a purified and well-characterized mAb to galectin-3 amino-terminal epitope, with the long half-life positron emitter zirconium-89 (^89^Zr; t_1/2_ = 74.8 h, β+ = 22.6%). Xenografted athymic Nude-Foxn1^nu/nu^ mice received 1.5 MBq (40 µCi) of ^89^Zr-labeled mAb to gal-3 in the tail vein and were subjected to imaging sessions at 48 h post-injection.

**Figure 3:**
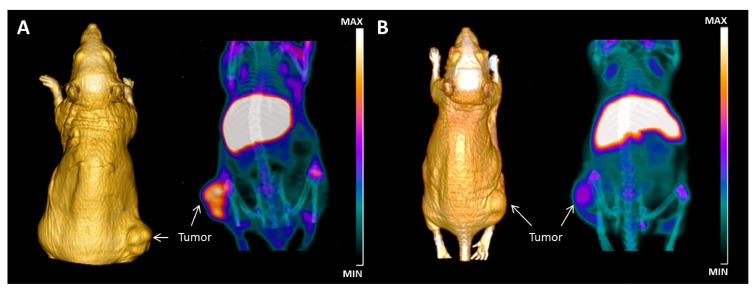
In vivo immuno-positron emission tomography (immuno-PET) imaging of thyroid tumor by targeting galectin-3. (**A**) Maximum intensity projection (MIP) of µ-PET images acquired at 48 h post-injection of 40 µCi of radiotracer showing accumulation of ^89^Zr-labelled mAb to gal-3 in subcutaneous tumor growing in the right thigh. 3D volume rendering (3D VR), based on CT acquisition performed using OsiriX Image Software 5.0 (Pixmeo, Geneva, Switzerland), allows a tri-dimensional reconstruction of the anatomy of the mouse and visualization of the tumor shape. The activity in the liver is due to residualization of antibody metabolites. (**B**) A strong reduction of uptake is visualized in mice pre-injected with 100-fold excess of unlabeled mAb to gal-3 and imaged 48 h post-injection of the radiotracer, confirming the binding specificity. Normal thyroid gland does not express detectable galectin-3, and as expected, no accumulation of the radiotracer was visible in the neck region.

Static PET/computer tomography (PET/CT) images showed high tumor binding specificity of the radiotracer and a reliable imaging of thyroid cancer in vivo [64] (Figure 3).

The reliability of the proposed imaging approach has been confirmed in three different animal models of human thyroid cancer xenografts including a follicular carcinoma and a poorly-differentiated thyroid carcinoma.

The specificity of galectin-3 immuno-PET targeting for imaging thyroid cancer has been further confirmed by an extensive ex vivo biodistribution analysis, measuring the amount of ^89^Zr-labeled probe accumulated in tumors and normal tissues explanted from the experimental animals [65].

Concluding, galectin-3 immuno-PET targeting represents a new potential diagnostic method for in vivo detection and biological characterization of thyroid nodules, which deserves to be further improved for clinical translation. Preclinical studies on orthotopic models of thyroid cancer are ongoing to confirm the preliminary findings. Galectin-3 immuno-PET strategy for imaging thyroid cancer in vivo is supported by a strong clinical and biological rationale and has the potential to improve, in the near future, the clinical management of patients bearing thyroid nodules, reducing unnecessary surgery and social costs [66,67,68].

Galectin-3 immuno-PET may potentially improve thyroid cancer diagnosis in the following conditions: (a) in the presence of multiple thyroid nodules, which cannot be easily analyzed preoperatively by using conventional FNA cytology; (b) in the presence of small suspicious sub-centimetric lesions (3–4 mm) discovered in a deep mediastinal position or intimately associated with vascular structures, for which fine-needle-aspiration-biopsy evaluation can be difficult or harmful; (c) the method may be also applied to distinguish among thyroid cancer infiltration/residual tumor nests and minimal normal thyroid tissue residues after thyroid surgery. This represents a common clinical problem during follow-up of patients after a thyroid cancer surgery. Although many of these conditions are presently detected with radioiodine, they remain largely uncharacterized at biological level.

(d) Moreover, a fraction of poorly-differentiated thyroid carcinomas and anaplastic thyroid carcinomas (rare lesions) generally express galectin-3, but lose the ability to uptake radioiodine.

In these specific cases, immuno-PET targeting of galectin-3 might be useful for detecting residual disease and to provide information for a better clinical management and therapeutic intervention of each specific case.

Although the preclinical experimental work performed with galectin-3 immuno-PET has been focused on thyroid cancer detection in vivo, the proposed technology could be useful in different tumor conditions. Galectin-3, in fact, is expressed in different primary and metastatic tumors (i.e., melanoma, breast carcinoma, prostatic carcinoma) [51]. The possibility to image these tumors in vivo or to detect metastasis (i.e., metastatic sentinel lymph nodes) represents an interesting field of research to be investigated.

At least theoretically, increasing the LET (linear energy transfer) of the radionuclide linked to the galectin-3-specific probe will generate a tool for immuno-radiotherapy and/or radio-ablation of the so-called “occult thyroid carcinomas” of millimetric dimension. These lesions are generally undetectable preoperatively. Further, preclinical studies in the field are ongoing, as well as humanization of galectin-3-specific mAbs and/or adequate structural modifications of these probes (i.e., the creation of radiolabeled F(ab)-fragments or chimeric molecules). This work will be necessary for translating the method to the clinical setting.

## 5. Conclusions

In the present Special Issue of the International Journal of Molecular Sciences focused on galectins in cancer and translational medicine, other experiences with galectin-1, galectin-3 and thyroid cancer are reported by different research groups [56,69]. Very interestingly, some papers published in this issue also show the possibility to inhibit specific galectins in vivo by using both natural and synthetic inhibitors [70]. We are confident that research in glycobiology of galectins will contribute in the near future to improve both cancer diagnosis and treatment of several tumor conditions, discovering targetable galectin-mediated functions that are critical for cancer [51,71].

Concluding: i) glycobiology of galectin-3; ii) the biological effects induced by galectin-3 in tumor cells in vitro; iii) the extensive characterization of galectin-3 expression performed on human thyroid tissues ex vivo; iv) the possibility to imaging thyroid cancer in vivo by using a galectin-3 immuno-PET represent all together a fashionable journey from the bench to the bed-side, which has already provided important improvements in clinical practice.

## Figures and Tables

**Table 1:** Technical requirements for galectin-3 expression analysis to be applied on fine-needle-aspiration-derived thyroid cells and conventional histological substrates.

A: A purified and well-characterized mAb to human galectin-3 (concentration ranging from 5–10 μg/mL) must be used in immunohisto-cytochemistry (direct or indirect immunoperoxidase) with a biotin-free detection system.
B: Galectin-3 immunostaining must be applied on formalin-fixed and paraffin embedded cyto-histological substrates (i.e., FNA-derived cellblocks).
C: Antigen retrieval microwave treatment with 0.01 M citrate buffer, pH6 for three cycles of 3–5 min each at 750 W is necessary.
D: Follicular thyroid cells showing galectin-3 accumulation in the cytoplasm, with or without nuclear staining, are considered positive. Scattered foamy macrophages serve as the internal positive control.
E: In Hashimoto’s thyroiditis (HT) and less frequently in chronic lymphocytic thyroiditis, false positive immunostaining for galectin-3 may occur in follicular thyroid cells within inflammatory follicles. This may generate false positive results (nodular lesions in HT always require a multidisciplinary clinical-pathological evaluation for a better therapeutic decision).
F: The surgical option for galectin-3-positive cases is advisable, also in the presence of a few galectin-3-positive thyroid follicular cells (cytoplasm +).

(Modified from Bartolazzi et al. [53].)
